# SARS-CoV-2 Seroprevalence Among Health Care Workers in Major Private and Public Hospitals With COVID-19 Patient's Referral in Tehran, Iran

**DOI:** 10.3389/fpubh.2022.832003

**Published:** 2022-03-24

**Authors:** Maryam Darvishian, Maryam Sharafkhah, Zahra Mohammadi, Khosro Sadeghniiat-haghighi, Alireza Abdollahi, Mohamadreza Jafary, Mona Talaschian, Payam Tabarsi, Parvaneh Baghai, Masoud Mardani, Amaneh Shayanrad, Fatemeh Shafighian, Melineh Markarian, Farzin Roozafzai, Mohammad Zamani, Saba Alvand, Sanam Hariri, Anahita Sadeghi, Hossein Poustchi, Reza Malekzadeh

**Affiliations:** ^1^Cancer Control Research, BC Cancer Research Centre, Vancouver, BC, Canada; ^2^Liver and Pancreatobiliary Diseases Research Center, Shariati Hospital, Digestive Diseases Research Institute, Tehran University of Medical Sciences, Tehran, Iran; ^3^Occupational Sleep Research Center, Tehran University of Medical Sciences, Tehran, Iran; ^4^Department of Pathology, School of Medicine, Imam Hospital Complex, Tehran University of Medical Sciences, Tehran, Iran; ^5^Shariati Hospital, Tehran University of Medical Sciences, Tehran, Iran; ^6^Department of Internal Medicine, Shariati Hospital, Tehran University of Medical Sciences, Tehran, Iran; ^7^Clinical Tb and Epidemiology Research Center, NRITLD, Shaheed Beheshti University of Medical Sciences, Tehran, Iran; ^8^Infectious Diseases and Tropical Medicine Research Center, Shahid Beheshti University of Medical Sciences, Tehran, Iran; ^9^Digestive Oncology Research Center, Digestive Diseases Research Institute, Shariati Hospital, Tehran University of Medical Sciences, Tehran, Iran; ^10^Digestive Disease Research Center, Digestive Diseases Research Institute, Shariati Hospital, Tehran University of Medical Sciences, Tehran, Iran

**Keywords:** SARS-CoV-2, seroprevalence, health-care worker, personal protective equipment, COVID-19

## Abstract

Estimating the prevalence of SARS-CoV-2 antibody seropositivity among health care workers (HCWs) is crucial. In this study, the seroprevalence of anti-SARS-CoV-2 antibodies among HCWs of five hospitals of Tehran, Iran with high COVID-19 patient's referrals from April to June, 2020, was assessed. In this cross-sectional study, HCWs from three public and two private hospitals, selected randomly as a pilot, were included. Participants were asked questions on their demographic characteristics, medical history, hospital role, and usage of personal protective equipment (PPE). Iran FDA-approved SARS-CoV-2 ELISA kits were used to detect IgG and IgM antibodies in blood samples. The seroprevalence was estimated on the basis of ELISA test results and adjusted for test performance. Among the 2,065 participants, 1,825 (88.4%) and 240 (11.6%) HCWs were recruited from public and private hospitals, respectively. A total of 340 HCWs were tested positive for SARS-CoV-2-specific IgG or IgM antibodies, and 17.9% of seropositive individuals were asymptomatic. The overall test performance-adjusted seroprevalence estimate among HCWs was 22.6 (95% CI: 20.2–25.1), and PPE usage was significantly higher among HCWs of public vs. private hospitals (66.5 vs. 20.0%). This study found that seroprevalence of SARS-CoV-2 among HCWs was higher in private hospitals (37.0%; 95% CI: 28.6–46.2) than public hospitals (20.7%; 95% CI: 18.2–23.3), and also highest among assistant nurses and nurses, and lowest among janitor or superintendent categories. The PPE usage was especially suboptimal among HCWs in private hospitals. Continued effort in access to adequate PPE and regular screening of hospital staff for detecting asymptomatic personnel, especially during the upcoming wave of infection, are warranted.

## Introduction

As the severe acute respiratory syndrome coronavirus 2 (SARS-CoV-2) reached a pandemic level, with more than 132 million cases globally by April 2021, the risk of virus transmission among health care workers (HCWs) with close contact with patients with coronavirus disease (COVID-19) has increased (1, 2). Hence, to reduce the risk of virus transmission and to assess safety precautions in hospitals, estimating the prevalence of antibody seropositivity among HCWs is crucial (3). Nevertheless, factors such as surge in COVID-19-related hospital admissions and limited access to diagnosis test could partly restrict the efforts in conducting seroprevalence surveys among HCWs (4, 5).

Overall, a higher prevalence of SARS-CoV-2 antibodies among HCWs compared to non-HCWs was reported in the studies conducted in different countries that include the USA and Sweden (2, 4, 6). Barret and colleagues found a significantly higher seroprevalence among HCW compared to non-HCW (7.3 vs. 0.4%), and about 62% of the positive subjects were nurses (4). In a large survey in USA on 10,275 HCWs previously trained for PPE use, 3.8% had positive serology for SARS-CoV-2 antibodies (7). In a longitudinal study, 1.2% of HCW were seropositive for SARS-CoV-2 antibodies in May 2020, whereas it increased to 4.6% in December, and the majority of them were among nurses and were men (8). Iran was among the first countries that reported widespread outbreaks of SARS-CoV-2 in several provinces (9). In a recently conducted study in 18 cities across 17 provinces in Iran, the difference in the prevalence of antibody seropositivity among frontline and non-frontline HCWs was low (21.6 vs.18.0%) (9). Similarly, in a study conducted among staff of Mofid children's hospital in Tehran, Iran, no difference was observed in the risk of seropositivity among HCWs vs. individuals working in administrative departments (10). The similar seroprevalence estimates among HCWs vs. non-HCWs in these studies could partly be due to the lack of compliance to safety protocols and/or limited access to personal protective equipment (PPE) in some hospitals (10, 11).

Although these studies provide some insights into the prevalence of SARS-CoV-2 antibodies among HCWs in Iran, their data did not include information on the potential variation in antibody seropositivity by HCW's hospital role, type of hospital (e.g., public vs. private), and hospital departments. Furthermore, although the use of PPE for reducing the risk of transmission in departments treating patients with COVID-19 has been recommended, it has been shown that by April 2020, 1,710 COVID-19 infections and 116 deaths among HCWs in Iran were related to insufficient access to PPE (11, 12). Hence, additional data on PPE protection against the prevalence of SARS-CoV-2 antibodies among HCWs in hospitals is required for work-safety policy decision-making.

To address the current knowledge gaps, in this study, we assessed the seroprevalence of anti-SARS-CoV-2 antibodies among HCWs in the five hospitals of Tehran, Iran, which had the most patients with COVID-19 in the first few months of the pandemic.

## Materials and Methods

### Ethical Statement

Both the study proposal and protocol were approved by the Ethics Committee of the Digestive Diseases Research Institute at Tehran University of Medical Sciences (reference number: IR.TUMS.DDRI.REC.1399.005).

### Study Design and Population

In this cross-sectional study, we used serological testing to assess the prevalence of SARS-CoV-2 antibodies among HCWs, in five hospitals in Tehran, Iran. We included government-based (i.e., public) teaching hospitals as they had major COVID-19 patient's referral in Tehran. We also included two randomly selected private hospitals, as pilot, from the listed private hospitals in Tehran.

Compared to the public hospitals, private hospitals are smaller and have lower number of HCWs. Hence, all HCWs who were working within two assigned days in the selected private hospitals were invited to participate in our study. In public hospitals, the required number of days for data collection was dependent on the total number of hospitals HCWs. Hence, total duration of data collection from the three public hospitals took 3, 2, and 3 days. Samples from those who agreed to participate were collected during the assigned data collection days, from April to June 2020.

### Test Characteristics

Detailed information on test characteristics was reported previously (9). In summary, Pishtaz Teb SARS-CoV-2 ELISA kits (catalog numbers PT-SARS-CoV-2.IgM-96 and PT-SARS-CoV-2.IgG-96) approved by Iran's Food and Drug Administration were used and validated to assess the presence of SARS-CoV-2-specific IgG and IgM antibodies in serum samples (9). The accuracy of the ELISA kits was validated using serum samples (collected within 2–4 weeks of symptom onset) from 154 patients with RT-PCR confirmed COVID-19, and 110 serum samples collected and stored in the Digestive Diseases Research Institute (DDRI) biobank, 2 years before the pandemic (9). Overall, 103/154 samples tested positive for either IgG [94 (61%)] or IgM [79 (51%)] with the ELISA kits, which resulted in sensitivity of 66.9% (95% CI: 58.9–74.2%) (9). Besides, 108/110 pre-pandemic samples tested negative for both IgG and IgM SARS-CoV-2-specific antibodies, which resulted in specificity of 98.2% (95% CI: 93.6–99.8) (9).

### Sample and Data Collection

Health care workers were categorized based on their occupation type as physicians, nurses, hospital technicians, administration staff, and janitor or building superintendents. Written informed consent was obtained from each individual. A unique barcode was given to each participant to label all biological samples and documentations. After informed consent was obtained, the HCWs underwent serology testing and their following information was collected: age, sex, and body mass index (BMI), the presence of comorbidity (i.e., the presence of at least one of the following conditions, namely, diabetes, heart disease, hypertension, lung disease, kidney disease, asthma, fatty liver disease, cirrhosis, hepatitis B, hepatitis C, HIV, autoimmune hepatitis, thalassaemia, hemophilia, dementia, multiple sclerosis, malignancy, inflammatory bowel disease, and history of organ transplantation), hospital type, contact with patients with COVID-19, PPE usage, categorized as mask only or mask and other equipment including gowns, shield, and/or goggles, HCW's hospital role, and working department. Furthermore, data on COVID-19-related symptoms were collected, and participants were categorized based on the experienced number of COVID-19-related symptoms into asymptomatic, paucisymptomatic (1–3 symptoms), or symptomatic (≥ 4 symptoms). The history of the COVID-19-related symptoms such as anosmia, sore throat, headache, shortness of breath, diarrhea, conjunctivitis, weakness, myalgia, arthralgia, altered level of consciousness, and chest pain in the preceding 12 weeks was also requested (9).

A laboratory technician then collected 5 ml of venous blood into an EDTA-coated microtainer which later were couriered to DDRI laboratory and stored at stored at −80°C (9). The detailed information on sample collection and ELISA kits has already been published elsewhere (9).

### Statistical Analysis

Baseline characteristics of participants among seronegative vs. IgG- or IgM-positive individuals were reported. Two-sided chi-squared test was used to compare categorical variables. To assess the seroprevalence of SARS-CoV-2-specific antibodies among HCWs, the overall crude frequencies of positive tests and test performance-adjusted estimates, stratified by age categories, sex, BMI, the presence of comorbidity, HCWs' job type, department of work, hospital type, diagnosed COVID-19, contact with infected patients, and symptom categories were estimated. The 95% confidence intervals (CIs) for crude seroprevalence were estimated using exact binomial models, and a bootstrap method was used to construct the 95% CIs for the adjusted estimates (9, 13). Logistic regression model was used to estimate the crude and adjusted odd ratios (ORs) and their 95% CIs for HCWs' job type, department of work, and hospital type. Full model was also adjusted for PPE usage and contact with patients with COVID-19. In the logistic models, the categories with lowest risk were considered as a reference. All statistical analyses were conducted by STATA software, version 12. The statistical approach is used for the test performance adjustment, and the bootstrap method is provided in detail elsewhere (9).

## Results

In total, among the 2,065 participants, 1,825 (88.4%) and 240 (11.6%) HCWs were recruited from the public and private hospitals, respectively (Table 1). Overall, 66.0% of participants were men, 41.4% aged 30–39 years, 52.1% had BMI ≤ 25, 24.3% had at least one comorbid condition, and 19.6% were working in COVID-19 patient ward (Table 1). Nurses and assistant nurses were the most and least frequent hospital roles (32.5 vs. 8.9%) among participants.

**Table 1:** Baseline characteristics.

	**Total** **(*N* = 2,065)** **n (%)**	**Seronegative** **(*N* = 1,725)** **n (%)**	**IgG or IgM positive (*N* = 340)** **n (%)**	***p-value***
Age (years), mean (SD)	37.49 (9.2)	37.29 (9.1)	38.46 (9.2)	0.033
Age categories, n (%)				0.232
<30	439 (21.7)	378 (22.4)	61 (18.3)	
30–39	839 (41.5)	704 (41.7)	135 (40.5)	
40–49	496 (24.5)	404 (23.9)	92 (27.6)	
50–59	222 (11)	184 (10.9)	38 (11.4)	
≥60	27 (1.3)	20 (1.2)	7 (2.1)	
Sex, n (%)				0.192
Female	702 (34)	576 (33.4)	126 (37.1)	
Male	1,363 (66)	1,149 (66.6)	214 (62.9)	
BMI, mean (SD)	25.17 (4.1)	25.09 (4.1)	25.55 (3.9)	0.105
BMI categories				0.014
≤ 25	1,058 (52.1)	909 (53.5)	149 (44.7)	
25.1–30	757 (37.3)	610 (35.9)	147 (44.1)	
>30	217 (10.7)	180 (10.6)	37 (11.1)	
Comorbidity, n (%)				0.719
No	1,557 (75.7)	1,303 (75.8)	254 (74.9)	
Yes	500 (24.3)	415 (24.2)	85 (25.1)	
Hospital type, n (%)				<0.001
Public	1,825 (88.4)	1,547 (89.7)	278 (81.8)	
Private	240 (11.6)	178 (10.3)	62 (18.2)	
Diagnosed COVID-19, n (%)				<0.001
No	313 (74.4)	267 (84.8)	46 (43.4)	
Yes	108 (25.7)	48 (15.2)	60 (56.6)	
Contact with patients with COVID-19, n (%)				0.008
No	433 (21)	380 (22)	53 (15.6)	
Yes	1,632 (79)	1,345 (78)	287 (84.4)	
Symptoms, n (%)				<0.001
Asymptomatic (0)	629 (30.6)	569 (33.1)	60 (17.9)	
Paucisymptomatic (1–3)	716 (34.9)	625 (36.4)	91 (27.2)	
Symptomatic (≥4)	708 (34.5)	524 (30.5)	184(54.9)	
Health worker positions				<0.001
Physicians	365 (17.7)	306 (17.8)	59 (17.4)	
Nurses	670 (32.5)	539 (31.3)	131 (38.5)	
Assistant nurses	184 (8.9)	145 (8.4)	39 (11.5)	
Janitor/building superintendents	349 (16.9)	316 (18.4)	33 (9.7)	
Hospital technicians	207 (10)	169 (9.8)	38 (11.2)	
Administration staff	286 (13.9)	246 (14.3)	40 (11.8)	
Health worker department				<0.001
COVID-19 patient ward	303 (19.6)	224 (17.2)	79 (32.5)	
ICU, or surgery ward	264 (17.1)	233 (17.9)	31 (12.8)	
Other wards	980 (63.4)	847 (65)	133 (54.7)	

In total, 340 HCWs were tested positive for SARS-CoV-2-specific IgG or IgM antibodies, 81.8% were employed at the public hospitals and 18.2% at the private hospitals. Overall, 17.9% of seropositive individuals were asymptomatic (Table 1).

In the analyses comparing PPE usage by hospital type, HCW's hospital role, and hospital department, combined usage of mask and any other type of PPE was significantly higher among HCWs of public hospitals than private hospitals (66.5 vs. 20.0%, *x*^2^ = 192.61, *p* = 0.000). Similarly, the usage of other PPE types that include gowns (46.9 vs. 20.4%, *x*^2^ = 39.24, *p* = 0.000), and shield and/or goggles (37.2 vs. 16.7%, *x*^2^ = 39.24, *p* = 0.000), were significantly higher in the public hospitals. Furthermore, combined usage of mask and any other type of PPE significantly varied among the HCW's hospital role, with the highest usage that was observed among nurses and the lowest among administrative staff (67.5 vs. 42.0%, *x*^2^ = 62.25, *p* = 0.000). Similarly, the frequency of combined mask and any other type of PPE usage significantly differed among hospital departments (ICU or surgery ward vs. COVID-19 patient ward vs. other wards: 75.4 vs. 72.6 vs. 62.9%, respectively, *x*^2^ = 20.26, *p* = 0.000) (Table 2).

**Table 2:** PPE usage among HCWs and hospitals.

	**Mask only**	**Mask and other type of PPE**	***p-value***
Health worker positions		226 (61.9)	<0.001
Physicians	139 (38.1)	452 (67.5)	
Nurses	218 (32.5)	123 (66.8)	
Assistant nurses	61 (33.2)	215 (64.2)	
Janitor/building superintendents	120 (35.8)	123 (55.7)	
Hospital technicians	98 (44.3)	120 (42.0)	
Administration staff	166 (58.0)		
Hospital type		220 (72.6)	<0.001
Public	612 (33.5)	199 (75.4)	
Private	192 (80.0)	616 (62.9)	
Health worker department			<0.001
COVID-19 patient ward	83 (27.4)	1,213 (66.5)	
ICU, or surgery ward	65 (24.6)	48 (20.0)	
Other wards	364 (37.1)	226 (61.9)	

Combined usage of mask and any other type of PPE vs. mask only showed significantly lower antibody seropositivity among nurses (17.0 vs. 24.8%, *x*^2^ = 5.59, *p* = 0.018) but not any other job categories.

The overall test performance-adjusted seroprevalence estimate among HCWs was 22.6 (95% CI: 20.2–25.1). Among hospital roles, the test performance-adjusted seroprevalence estimates were highest among assistant nurses (29.8; 95% CI: 21.1–40.0) and nurses (27.3; 95% CI: 22.8–32.2) and lowest among janitor or superintendent categories (11.8; 95% CI: 7.4–17.3) (Table 3). Also, the seroprevalence of SARS-CoV-2 was higher in private hospitals (37.0%; 95% CI: 28.6–46.2) compared to the public hospitals (20.7%; 95% CI: 18.2–23.3) and was higher in the COVID-19 patient ward (37.3; 95% CI: 29.9–45.5) (Table 3). The impact of hospital type, HCW's hospital role, and hospital department were not changed considerably in the logistic model even after adjusting for PPE usage and contact with infected patients. Besides, assistant nurses had highest odds of positive seroprevalence compared to the janitor or superintendent (2.29; 95% CI: 1.4–3.8), and private hospitals had significantly higher odds of seropositivity compared to the public hospitals (1.61; 95% CI: 1.1–2.3) (Table 4). Finally, the highest age-stratified test performance-adjusted seroprevalence was observed among HCWs aged ≥60 years (37.1; 95% CI: 14.3–68.4), with BMI 25.1–30.0 (27.1; 95% CI: 22.9–31.7), those in close contact with infected patients (24.3; 95% CI: 21.5–27.3), and symptomatic individuals (37.2; 95% CI: 32.3–42.4) (Table 5).

**Table 3:** Frequencies and prevalence of seropositive tests stratified according to health worker hospital role, department, and hospital type.

	**Frequencies**	**Prevalence (95%CI)**	**Test performance–adjusted prevalence (95%CI)**
Overall	340/2,065	16.5 (14.9–18.1)	22.6 (20.2–25.1)
Health worker positions			
Physicians	59/365	16.2 (12.5–20.3)	22.1 (16.5–28.5)
Nurses	131/670	19.6 (16.6–22.8)	27.3 (22.8–32.2)
Assistant nurses	39/184	21.2 (15.5–27.8)	29.8 (21.1–40)
Janitor/building	33/349	9.5 (6.6–13.0)	11.8 (7.4–17.3)
superintendents			
Hospital	38/207	18.4 (13.3–24.3)	25.5 (17.7–34.6)
technicians			
Administration	40/286	14.0 (10.2–18.6)	18.8 (12.9–25.8)
staff			
Hospital department			
COVID-19	79/303	26.07(21.2–31.4)	37.34(29.9–45.5)
patient ward			
ICU, or surgery	31/264	11.74(8.1–16.3)	15.30(9.7–22.2)
ward			
Other wards	133/980	13.57(11.5–15.9)	18.11(14.9–21.7)
Hospital type			
Public	278/1,825	15.23(13.6–17)	20.67(18.2–23.3)
Private	62/240	25.83(20.4–31.9)	36.97(28.6–46.2)

**Table 4:** Crude and adjusted odd ratios for the outcome of seropositive tests.

	**Crude OR (95% CI)**	***p*-value**	Adjusted^*^OR (95% CI)	***p*-value**
Health worker positions				
Physicians	1.85 (1.2–2.9)	0.008	1.70 (1.1–2.7)	0.024
Nurses	2.33 (1.6–3.5)	<0.001	2.10 (1.4–3.2)	<0.001
Assistant nurses	2.58 (1.6–4.3)	<0.001	2.29 (1.4–3.8)	0.001
Janitor/building	Ref.	—	Ref.	—
superintendents				
Hospital	2.15 (1.3–3.6)	0.003	2.17 (1.3–3.6)	0.003
technicians				
Administration	1.56 (0.9–2.5)	0.077	1.57 (0.9–2.6)	0.074
staff				
Hospital department				
COVID−19	2.65 (1.7–4.2)	<0.001	2.48 (1.6–3.9)	<0.001
patient ward				
ICU, or surgery	Ref.	—	Ref.	—
ward				
Other wards	1.18 (0.8–1.8)	0.436	1.22 (0.8–1.9)	0.353
Hospital type				
Public	Ref.	—	Ref.	—
Private	1.94 (1.4–2.7)	<0.001	1.61 (1.1–2.3)	0.007

* *Adjusted for PPE usage and contact with patients with COVID-19 as well as other variables in the table*.

**Table 5:** Frequencies and prevalence of seropositive tests stratified according to baseline characteristics.

	**Frequencies**	**Prevalence (95%CI)**	**Test performance-adjusted prevalence (95%CI)**
Sex			
Female	126/702	17.95(15.2–21)	24.84(20.6–29.5)
Male	214/1363	15.7(13.8–17.7)	21.39(18.5–24.5)
Age categories			
<30	61/439	13.9(10.8–17.5)	18.61(13.8–24.1)
30–39	135/839	16.09(13.7–18.8)	21.99(18.3–26.1)
40–49	92/496	18.55(15.2–22.3)	25.77(20.7–31.5)
50–59	38/222	17.12(12.4–22.7)	23.56(16.3–32.2)
≥60	7/27	25.93(11.1–46.3)	37.12(14.3–68.4)
BMI categories			
≤ 25	149/1,058	14.08(12–16.3)	18.9(15.8–22.3)
25.1–30	147/757	19.42(16.7–22.4)	27.11(22.9–31.7)
>30	37/217	17.05(12.3–22.7)	23.46(16.2–32.2)
Comorbidity			
No	254/1,557	16.31(14.5–18.2)	22.33(19.6–25.3)
Yes	85/500	17.0(13.8–20.6)	23.38(18.5–28.9)
Diagnosed COVID−19			
No	46/313	14.7(11–19.1)	19.84(14.1–26.6)
Yes	60/108	55.56(45.7–65.1)	82.7(67.5–97.4)
Contact with patients with COVID−19			
No	53/433	12.24(9.3–15.7)	16.06(11.5–21.4)
Yes	287/1,632	17.59(15.8–19.5)	24.29(21.5–27.3)
Symptoms			
Asymptomatic	60/629	9.54(7.4–12.1)	11.91(8.6–15.9)
(0)			
Paucisymptomatic	91/716	12.71(10.4–15.4)	16.78(13.2–20.9)
(1–3)			
Symptomatic	184/708	25.99(22.8–29.4)	37.21(32.3–42.4)
(≥4)			

## Discussion

In this cross-sectional study among HCWs, the frequency of PPE usage as well as seroprevalence of SARS-CoV-2 varied considerably by hospital type, hospital department, and HCW's hospital role. Overall, the highest prevalence of seropositivity was observed in the private hospitals, COVID-19 patients ward department, nurses and nurse assistants, and individuals aged 60 years and older. Furthermore, concurrent usage of mask and any other type of PPE was significantly higher among HCWs of public hospitals, ICU or surgery ward, and nurses. Finally, 17.6% (60/340) of participants who had positive test results for SARS-CoV-2 antibodies did not report experiencing any symptoms.

In general, our overall test performance-adjusted SARS-CoV-2 seroprevalence estimate of 22.5% among HCWs in private and public hospitals was similar to the reported seroprevalence estimates in conducted cross-sectional studies of HCWs in the UK (24.4%) (14), New York City (27.0%), and Saudi Arabia (26.5%) (2, 14, 15). Consistent with other studies, we also observed variation in SARS-CoV-2 seropositivity by HCWs' hospital role and department (4, 14). The highest seroprevalence was observed among nurses and assistant nurses with more than two times higher than janitors and also higher than physicians, which could be due to closer contact with patients infected with COVID-19 (4). A study in Italy found a triple odds of positive serology among nurses and nurse assistants compared to non-HCWs (16). Similarly a systematic review on 49 similar studies reported higher seropositivity in HCWs working in COVID-19 patient wards, direct work with patients, front lines, and health care assistants (17). Another study in middle east also reported that HCWs who spent a longer duration working with patients with COVID-19 were at increased risk for seropositivity (15). A large cross-sectional study on 1,40,782 HCWs in various hospital roles demonstrated higher odds of positive serology among internal medicine specialists and sub-specialities and lower odds of seropositivity among pathologists and forensic medicine specialist. Nurses and nurse assistants were also at the highest risk of positivity in this study (18). In contrast, antibody positivity in our study was lower among janitor or building superintendents, who were compared to nurses, may follow different hospital policies with respect to SARS-CoV-2 safety precautions (4, 19). For instance, according to the World Health Organization (WHO) rational use of PPE, the type of PPE that should be used among hospital cleaners who enter the room of patients with COVID-19 partly differs from what HCWs should use (e.g., using heavy-duty gloves) (20).

We observed 17.6% positive SARS-CoV-2 antibodies among HCWs with no history of COVID-19 symptoms. Similarly, several studies reported the same findings, which indicate the potential virus transmission among HCWs within hospital departments (14, 21, 22). In a study conducted in multistate hospital network in the USA, 29% of participants with detected antibodies reported no symptoms of COVID-19 (22). Besides, 3.4% of asymptomatic HCWs had a definite or borderline positive result in Canada (23). These findings highlight the potential ‘subclinical nature' of COVID-19 disease spectrum and the importance of testing HCWs regularly to prevent the virus spread within the hospital environment (21–24). An extreme use of PPE like as consistent usage of N95 mask and eye protection could be the reason for not having symptoms in HCWs with positive serology (25). In addition to screening HCWs for SARS-CoV-2 seropositivity, assessing viral load among asymptomatic and symptomatic participants could provide some information about the viral transmission and pathogenesis of SARS-CoV-2 in hospital setting (2).

In this study, we found that overall, the combined usage of mask and any other type of PPE among HCWs of public hospitals was significantly higher than private hospitals. As a result, the seroprevalence of SARS-CoV-2 was higher in private hospitals compared to the public hospitals even after adjusting for PPE usage (OR (95% CI): 1.61 (1.1–2.3). The observed difference in PPE usage could partly be attributed to the fact that the included public hospitals in this study were the major COVID-19 referral centers in Tehran. Hence, limited access to PPE supply in country may have caused unequal distribution of PPE in private vs. public hospitals. Furthermore, different policy and health regulations on PPE usage across private and public hospitals may contribute to the observed difference (4). Further investigation on potential impact of PPE shortage on infection transmission in private hospitals is required.

Considering the higher seroprevalence of SARS-CoV-2 among HCWs with BMI ≥ 25.1 and the fact that about 24% of our study participants had at least one comorbid condition, the risk of COVID-19 and its complications could be elevated among vulnerable hospital staff. On the other hand, since the risk of infection is higher among individuals with comorbidity condition, the viral transmission may also be higher among HCWs with underlying medical diseases (26–28). Hence, sufficient access to PPE as well as assigning HCWs with comorbid conditions to hospital wards with lower risk of infection may need to be considered as the potential strategies to reduce the risk of infection and mortality among HCWs (3).

To the best of our knowledge, this is the first seroprevalence study in Iran that reports the SARS-CoV-2 seropositivity among HCWs by hospital types, department, and participant's role. Additionally, the prevalence of seropositive tests was stratified according to the HCW's baseline characteristics and PPE usage. Despite the strengths, this study had some limitations that should be considered. First, since patients were recruited during short period of time in each hospital, the study participants may not be representative of all HCWs working in each center. This limitation may also occur since the two included private hospitals were randomly selected as a pilot, and hence, the findings may not be generalisable to all private hospitals in Tehran. Further investigation by including more hospitals in future studies is warranted. Second, data on baseline characteristics, the presence of COVID-19 symptoms, and contact with infected patients were collected using a self-reported questionnaire, which may introduce recall and/or misclassification bias in the study. Finally, among seropositive HCWs, it was not possible to differentiate between the community-acquired and hospital-transmitted infections. Hence, the potential routs of SARS-CoV-2 transmission among HCWs remains unknown.

In conclusion, the findings of this study imply that seroprevalence of SARS-CoV-2 among HCWs depends on hospital type, hospital department, and hospital role. The PPE usage, as a main strategy in infection prevention, was suboptimal, especially among HCWs in private hospitals. HCWs with close contact with patients with COVID-19 and with comorbidity conditions are at higher risk of infection, and continued effort in access to adequate PPE, regular screening of hospital staff for detecting asymptomatic personnel, to reduce transmissions within hospitals, especially during the upcoming wave of infection, are warranted (6).

## Data Availability Statement

The raw data supporting the conclusions of this article will be made available by the authors, without undue reservation.

## Ethics Statement

The studies involving human participants were reviewed and both the study proposal and protocol were approved by the Ethics Committee of the Tehran University of Medical Sciences (Reference Number: IR.TUMS.DDRI.REC.1399.005). The Ethical Committee performed the approval anonymously. The patients/participants provided their written informed consent to participate in this study. The patients/participants provided their written informed consent to participate in this study.

## Author Contributions

MD, ZM, KS-h, AA, MJ, MT, PT, PB, MMard, ASh, and FS contributed to the conceptualization. MS, PB, MMard, and ASh performed the analysis. MD, MS, and ZM drafted, revised, and prepared the manuscript. MD, MS, ZM, KS-h, AA, MJ, MT, PT, PB, MMard, ASh, FS, MMark, FR, MZ, SA, SH, ASa, HP, and RM took part in reviewing the manuscript. MD, MS, ZM, MMark, FR, MZ, SA, SH, and ASa finalized and prepared the manuscript for submission. HP and RM provided the resources and supervised the project. All authors contributed to the article and approved the submitted version.

## Funding

This research received funding from Iranian Ministry of Health and Medical Education (99-1-97-47932).

## Conflict of Interest

The authors declare that the research was conducted in the absence of any commercial or financial relationships that could be construed as a potential conflict of interest.

## Publisher's Note

All claims expressed in this article are solely those of the authors and do not necessarily represent those of their affiliated organizations, or those of the publisher, the editors and the reviewers. Any product that may be evaluated in this article, or claim that may be made by its manufacturer, is not guaranteed or endorsed by the publisher.

## References

[1] Siegel RL Miller KD Fuchs HE Jemal A Cancer statistics 2021'. CA Cancer J Clin . (2021) 71:7–33. 10.3322/caac.21654 33433946

[2] Venugopal U Jilani N Rabah S Shariff MA Jawed M Mendez Batres A. SARS-CoV-2 seroprevalence among health care workers in a New York City hospital: a cross-sectional analysis during the COVID-19 pandemic. Int J Infect Dis. (2021) 102:63–9. 10.1016/j.ijid.2020.10.036 33075539 PMC7566823

[3] Adams JG Walls RM. Supporting the health care workforce during the COVID-19 global epidemic. JAMA. (2020) 323:1439–40. 10.1001/jama.2020.3972 32163102

[4] Barrett ES Horton DB Roy J Gennaro ML Brooks A Tischfield J. 'Prevalence of SARS-CoV-2 infection in previously undiagnosed health care workers in New Jersey, at the onset of the U.S. COVID-19 pandemic. BMC Infect Dis. (2020) 20:853. 10.1186/s12879-020-05587-2 33198725 PMC7668027

[5] Khalagi K Safoora G Davood K Mohammad Ali M Siamak Mirab S Saeide A. Prevalence of COVID-19 in Iran: results of the first survey of the Iranian COVID-19 serological surveillance program. (2021) 2021:03.12.21253442. 10.1101/2021.03.12.21253442

[6] Rudberg AS Havervall S Månberg A Jernbom Falk A Aguilera K Ng H. SARS-CoV-2 exposure, symptoms and seroprevalence in healthcare workers in Sweden. Nat Commun. (2020) 11:5064. 10.1038/s41467-020-18848-0 33033249 PMC7544689

[7] Baker JM Nelson KN Overton E Lopman BA Lash TL Photakis M. et al. Quantification of occupational and community risk factors for SARS-CoV-2 seropositivity among health care workers in a large US health care system. Ann Intern Med. (2021) 174:649–54. 10.7326/M20-7145 33513035 PMC7877798

[8] Tomczyk S Hönning A Hermes J Grossegesse M Hofmann N Michel J. Longitudinal SARS-CoV-2 seroepidemiological investigation among healthcare workers at a tertiary care hospital in Germany. BMC Infect Dis. (2022) 22:80. 10.1186/s12879-022-07057-3 35073863 PMC8784861

[9] Poustchi H Darvishian M Mohammadi Z Shayanrad A Delavari A Bahadorimonfared A. SARS-CoV-2 antibody seroprevalence in the general population and high-risk occupational groups across 18 cities in Iran: a population-based cross-sectional study. Lancet Infect Dis. (2021) 21:473–81. 10.1016/S1473-3099(20)30858-6 33338441 PMC7833828

[10] Armin S Karbasian F Hoseinialfatemi SM Mansour Ghanaie R Rafiei Tabatabaei S Fahimzad SA. Revalence of SARS-CoV-2 specific antibodies in the staff of a children's hospital, in Tehran, Iran, Jundishapur. J Microbiol. (2020) 13:e108592. 10.5812/jjm.108592

[11] Dodangeh M. Iranian healthcare system against COVID-19', GERMS, 10: 112-14.Efron, B. 1979. Bootstrap methods: another look at the Jackknife', 7 %J. The Annals of Statistics. (2020) 1–26. 10.18683/germs.2020.1192 PMC7330514 32656108

[12] Park W Chawla A O'Reilly EM. Pancreatic cancer: a review. JAMA. (2021) 326:851–62. 10.1001/jama.2021.13027 34547082 PMC9363152

[13] Efron B. Bootstrap methods: Another look at the jackknife. Ann Statist. (1979) 7:1–26. Available online at: http://jeti.uni-freiburg.de/studenten_seminar/stud_sem_SS_09/EfronBootstrap.pdf (accessed May 13, 2008).

[14] Shields A Faustini SE Perez-Toledo M Jossi S Aldera E Allen JD. SARS-CoV-2 seroprevalence and asymptomatic viral carriage in healthcare workers: a cross-sectional study. Thorax. (2020) 75:1089–94. 10.1136/thoraxjnl-2020-215414 32917840 PMC7462045

[15] Amer HA Abdallah HA Alkheledan HS Alzarzour SH Shrahily A Tamim H. SARS-CoV-2 antibody prevalence among healthcare workers: A cross-sectional study at a quaternary healthcare center in Saudi Arabia. J Infect Public Health. (2022) 15:343–8. 10.1016/j.jiph.2022.01.018 35167996 PMC8802145

[16] Modenese A Casolari L Rossi G Della Vecchia E Glieca F D'Elia C. Factors Associated with SARS-CoV-2 Infection Risk among Healthcare Workers of an Italian University Hospital. Healthc Basel Switz. (2021) 9:1495. 10.3390/healthcare9111495 34828540 PMC8622462

[17] Galanis P Vraka I Fragkou D Bilali A Kaitelidou D. Seroprevalence of SARS-CoV-2 antibodies and associated factors in healthcare workers: a systematic review and meta-analysis. J Hosp Infect. (2021) 108:120–34. 10.1016/j.jhin.2020.11.008 33212126 PMC7668234

[18] Poletti P Tirani M Cereda D Guzzetta G Trentini F Marziano V. Seroprevalence of and risk factors associated with SARS-CoV-2 infection in health care workers during the early COVID-19 pandemic in Italy. JAMA Netw Open. (2021) 4:e2115699. 10.1001/jamanetworkopen.2021.15699 34228126 PMC8261609

[19] Vahidy FS Bernard DW Boom ML Drews AL Christensen P Finkelstein J. Prevalence of SARS-CoV-2 infection among asymptomatic health care workers in the Greater Houston, Texas, Area. JAMA Netw Open. (2020) 3:*e*2016451. 10.1001/jamanetworkopen.2020.16451 PMC12011328 32716512

[20] Walter FM Mills K Mendonça SC Abel GA Basu B Carroll N. Symptoms and patient factors associated with diagnostic intervals for pancreatic cancer (SYMPTOM pancreatic study): a prospective cohort study. Lancet Gastroenterol Hepatol. (2016) 1:298–306. 10.1016/S2468-1253(16)30079-6 28404200 PMC6358142

[21] Hains DS Schwaderer AL Carroll AE Starr MC Wilson AC Amanat F. Asymptomatic seroconversion of immunoglobulins to sars-cov-2 in a pediatric dialysis unit. JAMA. (2020) 323:2424–5. 10.1001/jama.2020.8438 32407440 PMC7226282

[22] Self WH Tenforde MW Stubblefield WB Feldstein LR Steingrub JS Shapiro NI. Seroprevalence of SARS-CoV-2 among frontline health care personnel in a multistate hospital network - 13 academic medical centers, April-June 2020. MMWR Morb Mortal Wkly Rep. (2020) 69:1221–6. 10.15585/mmwr.mm6935e2 32881855 PMC7470460

[23] Ferreira VH Chruscinski A Kulasingam V Pugh TJ Dus T Wouters B. Prospective observational study and serosurvey of SARS-CoV-2 infection in asymptomatic healthcare workers at a Canadian tertiary care center. PLoS ONE. (2021) 16:e0247258. 10.1371/journal.pone.0247258 33592074 PMC7886177

[24] Black JRM Bailey C Przewrocka J Dijkstra KK Swanton C. 'COVID-19: the case for health-care worker screening to prevent hospital transmission'. Lancet. (2020) 395:1418–20. 10.1016/S0140-6736(20)30917-X 32305073 PMC7162624

[25] Zhang S Guo M Wu F Xiong N Ma Y Wang Z. Factors associated with asymptomatic infection in health-care workers with severe acute respiratory syndrome coronavirus 2 infection in Wuhan, China: a multicentre retrospective cohort study. Clin Microbiol Infect Off Publ Eur Soc Clin Microbiol Infect Dis. (2020) 26:1670–5. 10.1016/j.cmi.2020.08.038 32911080 PMC7476563

[26] Martin C Montesinos I Dauby N Gilles C Dahma H Van Den Wijngaert S. Dynamics of SARS-CoV-2 RT-PCR positivity and seroprevalence among high-risk healthcare workers and hospital staff'. J Hosp Infect. (2020) 106:102–6. 10.1016/j.jhin.2020.06.028 32593608 PMC7316468

[27] Yang J Zheng Y Gou X Pu K Chen Z Guo Q. Prevalence of comorbidities and its effects in patients infected with SARS-CoV-2: a systematic review and meta-analysis. Int J Infect Dis. (2020) 94: 91–95. 10.1016/j.ijid.2020.03.017 PMC7194638 32173574

[28] Reilev M Kristensen KB Pottegård A Lund LC Hallas J Ernst MT. 'Characteristics and predictors of hospitalization and death in the first 11 122 cases with a positive RT-PCR test for SARS-CoV-2 in Denmark: a nationwide cohort. Int J Epidemiol. (2020) 49:1468–81. 10.1093/ije/dyaa140 32887982 PMC7499657

